# Mechanistic insights into dissolution enhancement of co-spray dried meloxicam with chitosan and solubilization agent

**DOI:** 10.1038/s41598-025-31245-1

**Published:** 2025-12-14

**Authors:** Tereza Vařilová, Petra Svačinová, Karl G. Wagner, Tomáš Pekárek, Petra Pullmannová, Jan Patera, Kristina Steffens, Oliver Macho, Barbora Vraníková, Zdenka Šklubalová

**Affiliations:** 1 Department of Pharmaceutical Technology, Faculty of Pharmacy, Charles University, Akademika Heyrovského 1203/8, Hradec Králové, 500 05 Czech Republic; 2 Department of Pharmaceutical Technology and Biopharmaceutics, University of Bonn, Gerhard-Domagk-Str. 3, 53121 Bonn, Germany; 3 Zentiva, k.s., U Kabelovny 130, 102 37 Prague 10, Czech Republic; 4 Skin Barrier Research Group, Faculty of Pharmacy, Charles University, Akademika Heyrovského 1203/8, Hradec Králové, 500 05 Czech Republic; 5 Department of Organic Technology, University of Chemistry and Technology Prague, Technická 5, Prague, 166 28 Czech Republic; 6 Institute of Process Engineering, Faculty of Mechanical Engineering STU, Bratislava, Námestie slobody 17, 812 31 Bratislava 1, Slovak Republic

**Keywords:** Co-spray drying, Dissolution enhancement, Meloxicam, Chitosan, Sodium lauryl sulphate, Chemistry, Materials science

## Abstract

This study presents a co-spray drying formulation approach to enhance the dissolution rate of meloxicam (MX), a poorly soluble model drug, by combining it with chitosan (CHIT) and sodium lauryl sulfate (SLS). Ternary formulations were prepared using both mini- and medium-scale spray dryers and compared with physical mixtures and RAW MX. In vitro dissolution testing revealed a significant increase in the release rate, with up to 80% of MX dissolved within 5 min. A comprehensive set of solid-state analytical techniques (XRPD, FTIR, and confocal Raman microscopy) revealed subtle changes in MX crystallinity and hydrogen-bonding interactions with both excipients. These findings support three key mechanisms contributing to enhanced dissolution: (i) reduced agglomeration of MX via adsorption onto the CHIT surface, (ii) improved wettability of products due to the presence of SLS, and (iii) increased matrix hydrophilicity facilitating drug–medium contact. The successful scale-up using a medium-scale spray dryer demonstrated the feasibility of this strategy for industrial application. The results highlight co-spray drying with functional excipients as a robust and scalable method for improving the biopharmaceutical performance of poorly soluble drugs. The simple co-spraying of the CHIT dispersion with the drug solution, without the need to dissolve the chitosan, is additional advantage.

**Supplementary Information:**

The online version contains supplementary material available at 10.1038/s41598-025-31245-1.

## Introduction

The bioavailability of the drug after oral administration is crucial in reaching the efficient concentration of an active substance in the systemic circulation. Water poorly soluble drugs may show a reduced bioavailability and later onset of therapeutic effect after oral administration despite the good permeability^1,2^. The solubility of such a drug can generally be improved by the preparation of the amorphous form, however, the main limitation is the compromised physical stability due to the high energetic state of this solid, which can lead to the recrystallization during the manufacturing process or the product storage^3,4^. For this reason, the amorphous form of a drug should be efficiently stabilized; the example includes the loading into mesoporous silica pores as recently demonstrated by Vraníková et al.^5^. Besides that, many further experimental research works and reviews focusing on a variety of techniques enhancing the drug solubility/dissolution rate have been published. The examples include the modification of the solid form to an alternative polymorph, preparation of co-crystals or solid dispersions, the use of cyclodextrins and the self-emulsifying systems as well as the specific preparation methods, such as the micellar solubilization, milling, hot melt extrusion, freeze drying, and spray drying^6–13^.

As pioneered by Kawashima et al.^14^ for salicylic acid, drug solubility can also be enhanced by spray drying. Recently, spray drying was also successfully used for vancomycin hydrochloride^15^, zolmitriptan^16^, or griseofulvin^17^. In spray drying, the feed solution or dispersion is atomized by a nozzle into a drying chamber, and the solvent is then vaporized by a drying gas resulting in the formation of solid particles of a product. This is frequently employed for simple drying operations in particle engineering as well as in the preparation of granules^8,18,19^. Currently, spray drying technique is also used for the preparation of co-processed excipients combining two or more excipients into one product with the improved compressibility properties^20^. Process (inlet temperature, feed flow rate, nozzle diameter, etc.) and formulation parameters (composition and properties of a liquid, dispersion type, and viscosity) have a major impact on the properties of final product, process reproducibility, and the yield^8,10,21–23^.

A relatively novel direction which can enhance the drug dissolution rate in the spray drying seems to be the combination of a suitable carrier and a surfactant as shown by Pomázi et al.^24^ for meloxicam (MX) mixture with polysorbate 80 and mannitol. MX is a non-steroidal anti-inflammatory drug (NSAID) widely used for the long-term treatment of chronic rheumatic disease^25^. It belongs to the enolic acid group based on Biopharmaceutical classification system (BCS IIa) exhibiting a poor aqueous solubility but the high permeability, reaching a maximum plasma concentration after few hours following oral administration^26^. To improve solubility/dissolution rate of MX, the spray drying with a carrier, the co-milling with a carrier or a surfactant, and the impregnation of the carrier have been already successfully employed^24,27–29^.

Chitosan (CHIT) is a deacetylated derivate of chitin, insoluble in water, which is a natural, biocompatible, non-toxic, biodegradable and mucoadhesive polysaccharide, extracted from the exoskeleton of invertebrates^30^. Physical and chemical properties and quality of CHIT depend on the degree of de-acetylation and the molecular weight^31^. The larger flat particles of CHIT allow the adhesion of the micronized MX onto its hydrophilic surface thus reducing agglomeration of primary particles, which improved the dissolution rate of MX by formation of interactive powder mixtures^27^.

The aim and novelty of this preformulation study is to enhance the dissolution rate of the poorly soluble model drug MX through the development of co-spray dried formulations combining a hydrophilic polymer carrier CHIT and an anionic surfactant (sodium lauryl sulfate, SLS). MX-loaded micelles were prepared in aqueous and ethanolic media and co-spray dried with dispersed CHIT. The dissolution performance of these ternary systems was compared with physical mixtures of the same components.

To elucidate the mechanisms underlying the improved dissolution, a comprehensive set of complementary analytical techniques was employed: differential scanning calorimetry (DSC), X-ray powder diffraction (XRPD), Fourier-transform infrared spectroscopy (FTIR), and confocal Raman microscopy to investigate thermal behavior, crystallinity, molecular interactions, and spatial distribution of components. The integrated use of these methods enabled detailed physicochemical and structural characterization of the co-processed systems, revealing subtle changes in MX solid-state properties and intermolecular interactions that contribute to enhanced dissolution.

## Methods

### Materials

The model drug meloxicam Form I (MX, Cadila Healthcare Ltd., Ankleshwar, India) and meloxicam Form III were obtained as gift samples from Zentiva, k. s. (Prague, Czech Republic). Chitosan (CHIT, JBICHEM International trading, Co., Ltd., Shanghai, China) of 85% deacetylation grade and viscosity of 30 mPa·s was used as a drug carrier. The surfactant sodium lauryl sulphate (SLS, Dr. Kulich Pharma, s. r. o., Hradec Králové, Czech Republic) was used for micellar solubilization. Sodium hydroxide (Penta s.r.o., Prague, Czech Republic), potassium dihydrogen phosphate (Dr. Kulich Pharma, s.r.o., Hradec Králové, Czech Republic) were used for dissolution testing, double distilled/ultrapure water for particle size measurement, and purified water, ethanol 96% v/v (technical grade, VWR Chemicals GmbH, Darmstadt, Germany) and methanol HPLC grade (Sigma-Aldrich, Prague, Czech Republic) as solvents.

### Preparation of samples

#### Determination of the critical micelle concentration of SLS

The critical micelle concentration (CMC) of the SLS aqueous solution was determined by tensiometer Krüss K100 (KRÜSS, Hamburg, Germany) using platinum ring detachment method (du Noüy´s) at 20 °C. The measurements were repeated three-times. The average value 0.3% (w/V) was observed. The CMC in ethanol was expected slightly higher due to the lower polarity of ethanol. The 3% (w/V) concentration of SLS was used to achieve the complete dissolution of MX in water before the spray drying; the same concentration was used also in ethanol.

#### Preparation of mixtures by spray drying

Three types of spray dried mixtures (SDM) were prepared. The samples were coded as follows: the aqueous system (SDMW) and/or ethanolic system (SDME) were dried in a small laboratory scale (“mini-scale”) spray dryer (B-290, Büchi Labortechnik AG, Flawil, Switzerland). The pilot scale-up aqueous sample (SDML) was dried using a laboratory scale (“medium-scale”) spray dryer (Niro atomizer D-400 Mobile Unit Minor, Niro Atomizer Ltd., Copenhagen, Denmark).

To prepare the samples, the powder MX and a surfactant SLS were first mixed in a mortar. Subsequently, purified water (SDMW or SDML samples) or ethanol (SDME sample) was added. The samples were stirred overnight on a magnetic stirrer (Cimarec i Multipoint 15, ThermoFisher Scientific, Waltham, MA, USA) to achieve a micellar solution of MX. Finally, CHIT was dispersed in the liquid sample. To ensure homogeneity, the suspension was continuously stirred on a magnetic stirrer during spray drying. The initial composition of mixtures and the ratio of substances is shown in Table 1.

**Table 1:** Composition of spray dried mixtures and physical mixtures.

Mini Büchi spray dryer			
	Mass of substances/250 mL of mixture		
	MX (mg)	CHIT (g)	SLS (g)
SDMW	62.50	12.50	7.50

Mini Büchi spray dryer with an inert loop and dehumidifier			
	Mass of substances/1,000 mL of mixture		
	MX (mg)	CHIT (g)	SLS (g)
SDME	200.00	40.00	24.00
MX-CHIT SDE	200.00	40.00	-
MX SDE	200.00	-	-

Laboratory spray dryer			
	Mass of substances/4,000 mL of mixture		
	MX (mg)	CHIT (g)	SLS (g)
SDML	1,000.00	200.00	120.00

Physical mixtures			
	Mass of substances		
	MX (mg)	CHIT (g)	SLS (g)
PM	62.50	12.50	-
SPM	62.50	12.50	7.50

The open-loop configuration (SDMW, SDML) using air as a drying medium which was not re-circulated, and the closed-loop (SDME) based upon recycling nitrogen throughout the entire process were used. During the spray drying, the dispersion was continuously stirred on a magnetic stirrer to prevent sedimentation of CHIT and nozzle clogging. The aqueous systems were dried at 170 °C while 85 °C was used for ethanolic dispersions according to the ethanol lower boiling point. The parameters of spray drying are listed in Supplementary Table S1.

Additionally, the ethanolic solutions of MX (MX SDE) and MX in a CHIT dispersion (MX-CHIT SDE, in the same ratio as SDME) were spray dried under the same conditions. The samples were collected and dried under a vacuum (0.01–0.02 bar) (VDL-23, Binder GmbH, Tuttlingen, Germany) at 40 °C for 24 h to remove the residual ethanol.

#### Preparation of physical mixtures

For the comparison, the binary physical mixtures of MX and CHIT (PM) as well as ternary mixtures with the addition of SLS (SPM) were prepared in the same initial ratio of substances (Table 1). All used powders were sieved through a 500 μm sieve, then MX was mixed with CHIT using a sandwich method^27^ by a 3D blender (Turbula type T2F, WAB AG, Muttenz, Switzerland) at 34 rpm for 5 min. By a rule of thumb, the container was always below half full to achieve homogeneity of the mixture. In SPMs, MX was first mixed in a porcelain mortar with SLS before mixing with CHIT.

### Characterization of raw materials and co-processed samples

#### Moisture content analysis

The moisture content was evaluated by loss-on-drying method (LOD, Moisture analyser XM60, Precisa, Dietikon, Switzerland). The appropriate mass of sample was uniformly spread onto an aluminium pan and dried at 105 °C until the constant weight was achieved. The measurements were repeated 3 times, the mean value and standard deviation (StD) were calculated.

#### Particle size distribution

Particle size and size distribution of the raw substances and all samples (SDM and PM) were determined by a laser diffraction (Mastersizer 3000 Laser Diffraction Analyzer, Malvern Instruments, Malvern, UK) by the dry cell method (Aero S unit) using Mie theory. Air pressure was set to 3.0 bar, feed rate to 80%. The particle refractive index was 1.72 for MX^32^, 1.52 for CHIT^33^, and 1.33 for SLS^34^. The refractive index 1.60 was used for measurements of the prepared mixtures. The particle size values of Dv_10_ (µm), Dv_50_ (µm) and Dv_90_ (µm), which correspond to 10%, 50%, and 90% of the cumulative volume distribution, were determined together with “span” value characterizing the width of particle volume size distribution. Three measurements were carried out; mean values and StD were calculated.

#### Scanning electron microscopy (SEM)

The granulometric characteristics of samples were determined by scanning electron microscope (Phenom Pro, Phenom-World B. V., Eindhoven, Netherlands) with the Back-scattered Electron Detector (BSE). A small amount of the sample was sprinkled on a carbon conductive tape and coated with an approx. 10-nm-thick gold layer (Quorum Techn., Ltd., Lewes, UK). The acceleration voltage of 5 kV or 10 kV was applied, and images were collected at a magnification of 500x and 2,000x.

#### Evaluation of drug content and homogeneity assessment

Five randomly selected samples (100.0 ± 0.2 mg) were dispersed for 15 min in 10 mL of methanol using ultrasonic bath (WUC-A01H, Witeg Labortechnik GmbH, Wertheim, Germany). After filtration through a glass fibre filter (pore size 2.7 μm, Whatman), 200 µL of the solution was diluted by phosphate buffer (pH 6.8) up to 2 mL. The absorbance of the sample was detected by a spectrophotometer (Specord 205, Analytic Jena AG, Jena, Germany) at 363 nm five times and the concentration of MX was calculated according to a calibration curve. Mean value (*n* = 5) and standard deviations (StD) were calculated.

#### In vitro dissolution

The dissolution rate of MX was determined by the USP-4 flow-through cell apparatus Sotax CE-1 (Sotax AG, Aesch, Switzerland) using an open-loop system at 37 ± 0.5 °C. The samples were sieved through a 500 μm sieve and filled into dissolution powder cell (12 × 32 mm). Raw MX (RAW MX), MX SDE, and MX-CHIT SDE were used as the reference samples.

To keep a constant mass of 0.5 mg of MX in each SDM sample, the equivalent mass of 374.57 mg (MX-CHIT SDE), 223.25 mg (SDMW), 239.60 mg (SDME), and 186.10 mg (SDML) were used. RAW MX was the only exception as 5.0 mg of MX was necessary to gain a detectable amount of MX released within 5 min of the dissolution experiment. The equivalent mass of 100.62 mg (PM) or 161.86 mg (SPM) was used for physical mixtures.

Phosphate buffer pH 6.8 was used as a dissolution medium; the flow rate was set to 22 mL/min by a piston pump Sotax CY 1–50 (Sotax AG, Aesch, Switzerland). The exiting samples were collected manually at a time interval of 20 s in the first three minutes and 60 s intervals for the remaining time until 5 min was achieved in total. The concentration of dissolved MX was determined spectrophotometrically.

The relative amount of dissolved (released) drug *m*_rel_ (%) and the relative dissolution rate *r*_*rel*_ (min^− 1^) were calculated using Eqs. (1) and (2),

1 $${m_{rel~}}=\frac{{\sum c \times Q \times t}}{{{m_{MX}}}} \times 100~$$

2 $${r_{rel}}=\frac{{c \times Q}}{{{m_{MX}}}}~$$

where *c* is the measured concentration at the outlet of the dissolution cell (mg/L), *Q* is the flow rate (L/s) of the dissolution medium, *t* is the sampling interval (s) and m_MX_ is the total mass (mg) of MX in the sample^27^. The dissolution experiments were repeated in triplicate. The mean values of *m*_rel_ (%) and *r*_rel_ (min^− 1^), and the StD were calculated.

#### Differential scanning calorimetry (DSC)

Thermal properties of the raw substances and all samples were evaluated by differential scanning calorimetry (DSC 200 F3 Netzsch Maia, Proteus, Netzsch-Geratebau GmhH, Selb, Germany). The appropriate mass of sample (7 ± 0.1 mg, MYA 5.4Y PLUS Microbalances, Radwag, Radom, Poland, d = 0.001 mg) was inserted into a standard aluminum pan with a pierced lid. The sample was then held isothermally under nitrogen atmosphere at a temperature of -20 °C for 5 min prior to the first heating to 280 °C and for 2 min before the second heating up to the same temperature; the heating rate 10 °C/min was used. The thermograms were evaluated using program DSC 200 F3 Netzsch Proteus.

#### X-ray powder diffraction analysis (XRPD)

Raw substances, and the selected samples MX SDE, MX-CHIT SDE, SDME, and SDML were evaluated by XRPD by laboratory XPERT PRO MPD (PANalytical, Almelo, Netherlands) diffractometer with an X’Celerator and nickel filtered CuKα radiation (λ = 1.5409 Å). The generator operated at excitation voltage 45 kV and anodic current 40 mA. The back-loading technique with a powder sample preparation kit (PW1770/10) with 16 mm sample holder was used; the reflection mode of X-rays was measured in the range of 10°–30° (2θ) at increments of 0.04°/s. The following scan parameters were utilized: scan type – gonio, step size 0.02° 2θ and time per step 200 s. The data were analysed using X’Pert High score plus software (PANalytical, Almelo, Netherlands).

#### Fourier transform infrared (FTIR) analysis

FTIR analysis was performed by a Spectrum Two FT-IR spectrometer (PerkinElmer, Waltham, MA, USA). A 5–10 mg powder sample (RAW MX, MX SDE) was pressed onto the crystal using a flat-tip clamp. Samples (12 scans) were analysed in the 450–4,000 cm^− 1^ range using PerkinElmer Spectrum (Waltham, MA, USA) system.

#### Confocal Raman microscopy

Raman spectra and optical images were obtained at room temperature using a confocal Raman microspectrometer Alpha 300R (WITec, Ulm, Germany). The excitation radiation wavelength of a diode/solid-state laser was 633 nm. The laser energy on the output was 30 mV. A spectrograph UHTS 300 with a thermoelectrically cooled charge-coupled device detector collected the spectra. Zeiss objectives (10x EC „Epiplan“ DIC, 100x EC „Epiplan-Neofluar“ DIC) and a 600 lines/mm diffraction gratin were used. The spectral resolution in the range 500–3,100 cm^− 1^ was 3.2 cm^− 1^/pixel. Single spectra were acquired with a 0.5 s integration time and 20 × accumulations. The area scans and depth profiles had the step width of 500 nm in the x and y direction and 1,000 nm in the z direction; the maximum spatial resolution in the x and y directions was 364 nm. Scan dimensions are indicated by scale bars in pertinent figures. Data analysis was performed using WITec Project FIVE + software; the individual spectral components were identified and demixed using True Component analyses software toolkit.

### Statistical analysis

Experimental data were processed in MS Excel. The one-way analysis of variance (ANOVA) followed by two sample t-test were used to evaluate the differences in maximum relative dissolution rate *r*_MAX_ of RAW MX and the other prepared samples with p values of < 0.05 considered significant. To visualize the differences between *r*_*MAX*_, ANOVA in program GraphPad Prism (10.1.2, GraphPad Software, CA) was used at level of significance *p* < 0.05.

## Results and discussion

### Characterization of raw materials and co-processed samples

Three spray-dried mixtures (SDMs) were prepared to enhance the dissolution of the poorly soluble model drug meloxicam (MX) using chitosan (CHIT) as a polymer carrier and sodium lauryl sulfate (SLS) as a solubilizer. Two SDMs were produced on a mini-scale spray dryer using aqueous (SDMW) or ethanolic (SDME) feed solutions, and one on a medium-scale device (SDML, aqueous). A fixed initial weight ratio of MX: SLS: CHIT was employed across all formulations and corresponding physical mixtures (Table 1), in line with previous findings on the importance of drug-to-carrier ratios^27,35^. Reference samples included RAW MX, spray-dried MX from ethanol (MX SDE), and MX co-spray-dried with CHIT (MX-CHIT SDE).

Moisture content of raw materials (MX: 1.2%, SLS: 7.2%, CHIT: 9.2%) can influence both process efficiency and product stability^36^. The residual moisture of the spray-dried samples ranged from 1.4% (MX SDE) to 7.8% (MX-CHIT SDE), with the lowest value in ternary SDMs observed for SDML (1.8%), likely due to more efficient drying at medium scale. In contrast, physical mixtures retained higher moisture (PM: 10.6%, SPM: 7.3%), reflecting the hygroscopic nature of the components.

Spray drying conditions were tailored to solvent properties: aqueous feeds were processed at 170 °C, while ethanolic systems required only 85 °C. Production yields varied significantly, with SDME and SDML achieving 72% and 62%, respectively, while SDMW suffered from low recovery (16%), limiting its further characterization.

#### Morphological characterization

**Fig. 1:**
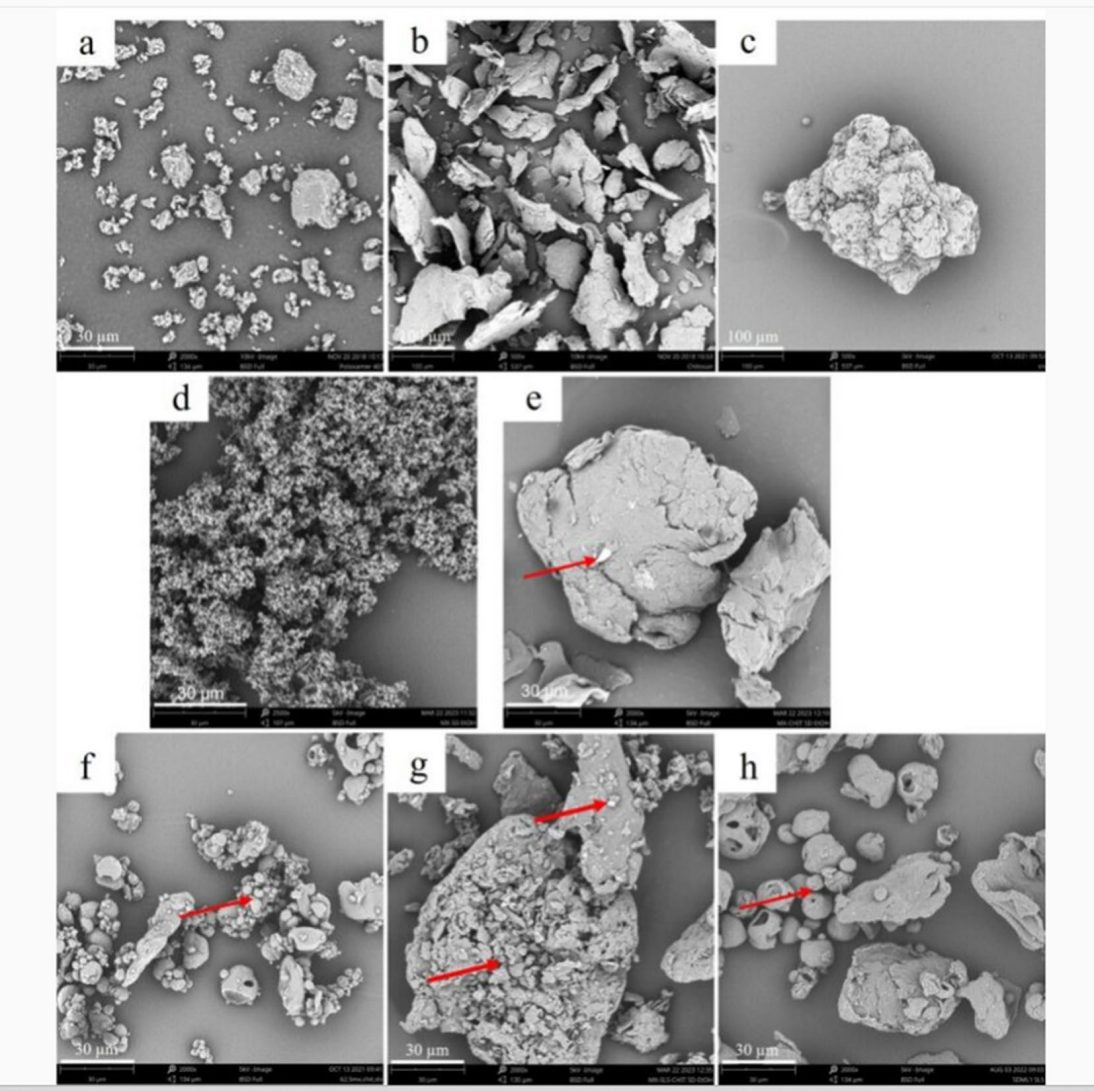
SEM Images of raw substances (a) RAW MX (magnification 2,000x), (b) CHIT (magnification 500x), (c) SLS (magnification 500x), (d) MX SDE, (e) MX-CHIT SDE, and spray dried mixtures (f) SDMW, (g) SDME, (h) SDML (magnification 2,000x). The inserted arrows show the MX particles and/or MX-SLS agglomerates.

Particle size distribution and morphology of raw materials and spray-dried samples are summarized in Table 2; Fig. 1. RAW MX exhibited a median particle size (Dv_50_) of 3.67 μm with a narrow distribution (span 1.95), while CHIT and SLS showed significantly larger Dv_50_ 60.93 μm and 190.33 μm, respectively, and broader distributions.

**Table 2:** Granulometric properties of meloxicam, chitosan, sodium Lauryl sulphate and the spray-dried samples.

Sample	Dv_10_ (µm)	Dv_50_ (µm)	Dv_90_ (µm)	Span (µm)
RAW MX	1.10 ± 0.00	3.67 ± 0.40	8.22 ± 0.02	1.95 ± 0.01
SLS	16.23 ± 1.63	190.33 ± 16.74	429.00 ± 13.00	2.18 ± 0.13
CHIT	19.97 ± 0.58	60.93 ± 0.92	157.67 ± 2.05	2.26 ± 0.02
MX SDE	0.69 ± 0.01	1.56 ± 0.03	6.08 ± 0.75	3.45 ± 0.43
MX-CHIT SDE	23.25 ± 0.21	61.95 ± 0.78	143.00 ± 1.41	1.93 ± 0.00
SDMW	3.35 ± 0.37	11.08 ± 1.44	40.73 ± 4.16	3.39 ± 0.18
SDME	4.53 ± 0.04	35.87 ± 1.07	137.00 ± 0.07	3.70 ± 0.07
SDML	8.80 ± 0.29	31.07 ± 0.75	112.33 ± 0.08	3.34 ± 0.08

The data are given as the mean ± StD.

Spray drying of MX from ethanol (MX SDE) yielded finer particles (Dv_50_ 1.56 μm), consistent with the lower surface tension of organic solvents. In contrast, ternary SDMs exhibited broader distributions and larger particle sizes, particularly SDME (Dv_50_ 35.87 μm) and SDML (Dv_50_ 31.07 μm), compared to SDMW (Dv_50_ 11.08 μm). These differences reflect the influence of feed rate, nozzle diameter, and spray dryer configuration^8,10,37–39^.

Despite the smaller nozzle 0.7 mm used in SDME vs. 1.4 mm in SDMW, the higher feed rate (11 mL/min vs. 2.5–3 mL/min) likely contributed to the formation of larger particles. The similarity in particle size between SDME and SDML, despite differing scales and nozzle types (two-fluid vs. rotary), further highlights the complexity of spray drying dynamics^38,39^.

SEM analysis (Fig. 1) revealed distinct morphological features. RAW MX formed small, irregular aggregates (Fig. 1a), while CHIT appeared as large, plate-like particles (Fig. 1b). SLS exhibited voluminous, irregular structures (Fig. 1c). MX SDE particles in Fig. 1d formed tiny irregular aggregates, whereas in MX-CHIT SDE, MX was distributed on the CHIT surface (Fig. 1e). Ternary SDMs showed varied morphologies: SDMW (Fig. 1f) and SDML (Fig. 1h) contained hollow spherical particles, likely due to rapid solvent evaporation at higher inlet temperatures^8,20,39^, while SDME (Fig. 1g) displayed shrunk, irregular particles, attributed to ethanol’s lower polarity and altered micelle behavior^40^.

Overall, particle size and morphology were strongly influenced by formulation composition, solvent properties, and spray drying parameters, all of which are critical for optimizing dissolution performance^41^.

#### In vitro dissolution study

**Fig. 2:**
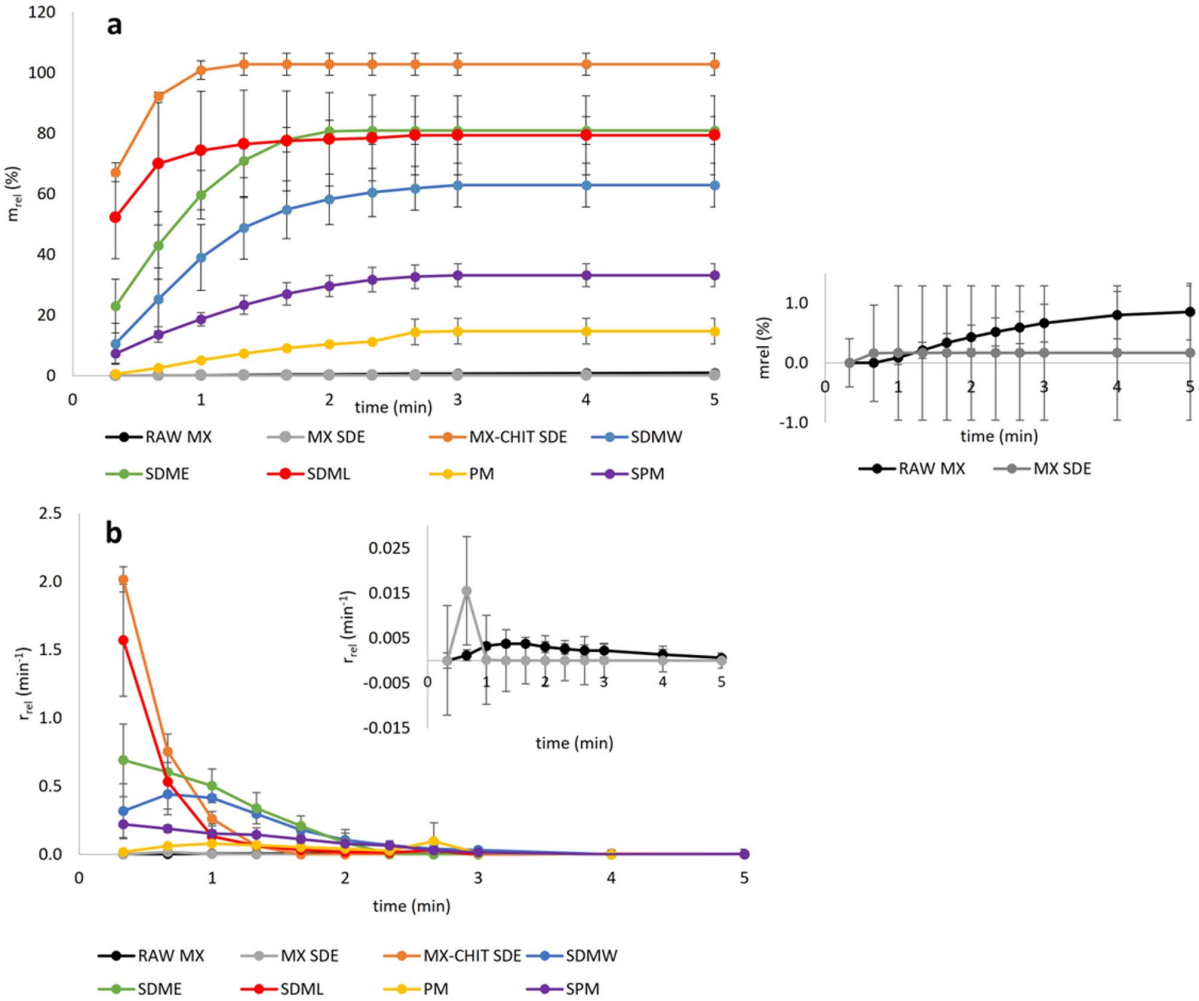
(a) Relative amount of released meloxicam *m*_rel_ (%) and (b) relative dissolution rate of meloxicam *r*_*rel*_ (min^− 1^) from the samples (RAW MX, spray dried samples and physical mixtures) within 5 min (*n* = 3); the inserted graphs show the zoomed dissolution profile of RAW MX and MX SDE.

An adequate amount of each powder sample was filled into the flow-through powder cell to ensure a constant MX mass of 0.5 mg per sample, based on the yield of embedded MX: 53.66 ± 2.4% (MX-CHIT SDE), 71.89 ± 2.8% (SDMW), 66.99 ± 1.7% (SDME), and 86.24 ± 3.5% (SDML). For comparison, PM and SPM yielded 100 ± 2.2% of MX, and equivalent masses of 100.62 mg (PM) and 161.86 mg (SPM) were used accordingly. The relative amount of released meloxicam (*m*_*rel*_, %) and the relative dissolution rate (*r*_*rel*_, min^–1^) for the reference samples, spray-dried formulations (SDMs), and physical mixtures are presented in Fig. 2.

As shown in Fig. 2a, RAW MX exhibited minimal dissolution (*m*_*rel*_ 0.86% at 5 min), consistent with its poor wettability and strong particle agglomeration^27^. In contrast, MX SDE showed significantly improved dissolution (*m*_*rel*_ 0.17%, *p* = 0.0002), with a higher initial dissolution rate (*r*_*rel*_ 0.02 min^–1^ vs. 0.0037 min^–1^ for RAW MX; Fig. 2b). However, both samples converged to similarly low rates after 5 min. It is essential to remind that the mass of the RAW MX sample equivalent to 5 mg of MX was necessary used to achieve detectable *m*_*rel*_.

MX-CHIT SDE achieved complete dissolution within 80 s, with a rapid initial rate (*r*_*rel*_ 2.01 min^–1^), highlighting the synergistic effect of CHIT as a hydrophilic carrier^27^. The dispersion of MX particles on the hydrophilic CHIT surface (Fig. 1e) likely enhanced wettability and facilitated medium penetration.

All ternary SDMs significantly improved MX dissolution compared to RAW MX (*p* = 0.014). Among mini-scale formulations, SDME (ethanolic) outperformed SDMW (aqueous), with *m*_*rel*_ 80.93% and *r*_*rel*_ 0.69 min^–1^ vs. 62.93% and 0.44 min^–1^, respectively. SDML (medium-scale) achieved comparable *m*_*rel*_ (79.38%) but a significantly higher *r*_*rel*_ (1.57 min^–1^, *p* = 0.0351), likely due to improved MX particle spreading on the CHIT surface and wettability (Fig. 1h). The enhanced performance of SDML over SDME, despite similar particle sizes, may be attributed to differences in particle morphology and surface accessibility. In SDME, MX and SLS formed dense agglomerates (Fig. 1g), while in SDML, MX was more evenly distributed on CHIT, facilitating faster release. In SDMW, partial CHIT swelling may have hindered MX diffusion^42,43^. Generally, higher error bars were observed at the beginning of dissolution. The powder bed retains air in the voids, which affects the initial contact of the dissolution medium with the powder particles and leads to higher data variability as visible in Fig. 2. The role of SLS in SDMs is twofold: it enhances wettability and forms micelles that encapsulate MX during atomization. Upon drying, the gas–liquid interface of the droplets becomes saturated with surfactant molecules, SLS accumulates at the droplet surface^44–46^, potentially coating MX particles and modulating their release. In an aqueous environment (SDMW sample in Fig. 2) where evaporation is slower, such a crust formed on the surface of some particles can affect the drug release rate and contribute to data variability. However, comparison SDME with MX-CHIT SDE suggests that SLS did not further enhance dissolution beyond the effect of CHIT alone. In aqueous media, SLS is essential for ensuring the solubility of MX. As the results showed, its effect is equivalent to that of ethanolic medium.

Physical mixtures showed moderate improvements (*m*_*rel*_ 15% for PM, 33% for SPM), confirming the de-agglomerating effect of CHIT and the wetting enhancement by SLS. Nevertheless, their performance remained inferior to that of co-processed SDMs, underscoring the importance of spray drying in achieving optimal drug dispersion and dissolution. In the SDM samples, free micronized MX particles located on the CHIT carrier surface are easily available for medium increasing the efficient sample surface. When such MX particles dissolve, dissolution rate becomes slower. The reduction in the efficient surface of MX in combination with SLS coating and the CHIT swollen particles can protect MX from the further dissolution^28^.

### Mechanistic analysis of co-processed samples

The results clearly proved the positive effect of the co-spray drying in the presence of SLS and CHIT on the dissolution of MX. However, the better dissolution of MX might be also based on the change in the MX crystalline structure. Therefore, to address the mechanism of the higher MX dissolution rate, DSC, XRPD, FTIR and Raman spectroscopy were used.

Five different crystal lattices (I - V) exhibiting distinct properties were described for MX^25,47,48^. The most common Form I popular in the pharmaceutical products for its stability, is unfortunately the least soluble in water. This form has been fully characterized; the characterization of remaining forms is impeded by troubles noted in their crystallization^47,49^. The metastable, more soluble polymorphs II and III convert to the Form I within a relatively short time; also, the Form V converts to Form I although it remains stable during the temperature range 130–190 °C for some time^48^. Recently, the Form IV is considered the crystal structure of MX hydrate, not a polymorph^25,47–49^.

#### Physico-chemical characterization

To elucidate the mechanism behind the enhanced dissolution of meloxicam (MX) in co-processed systems, differential scanning calorimetry (DSC), X-ray powder diffraction (XRPD), and Fourier-transform infrared spectroscopy (FTIR) were employed.

**Fig. 3:**
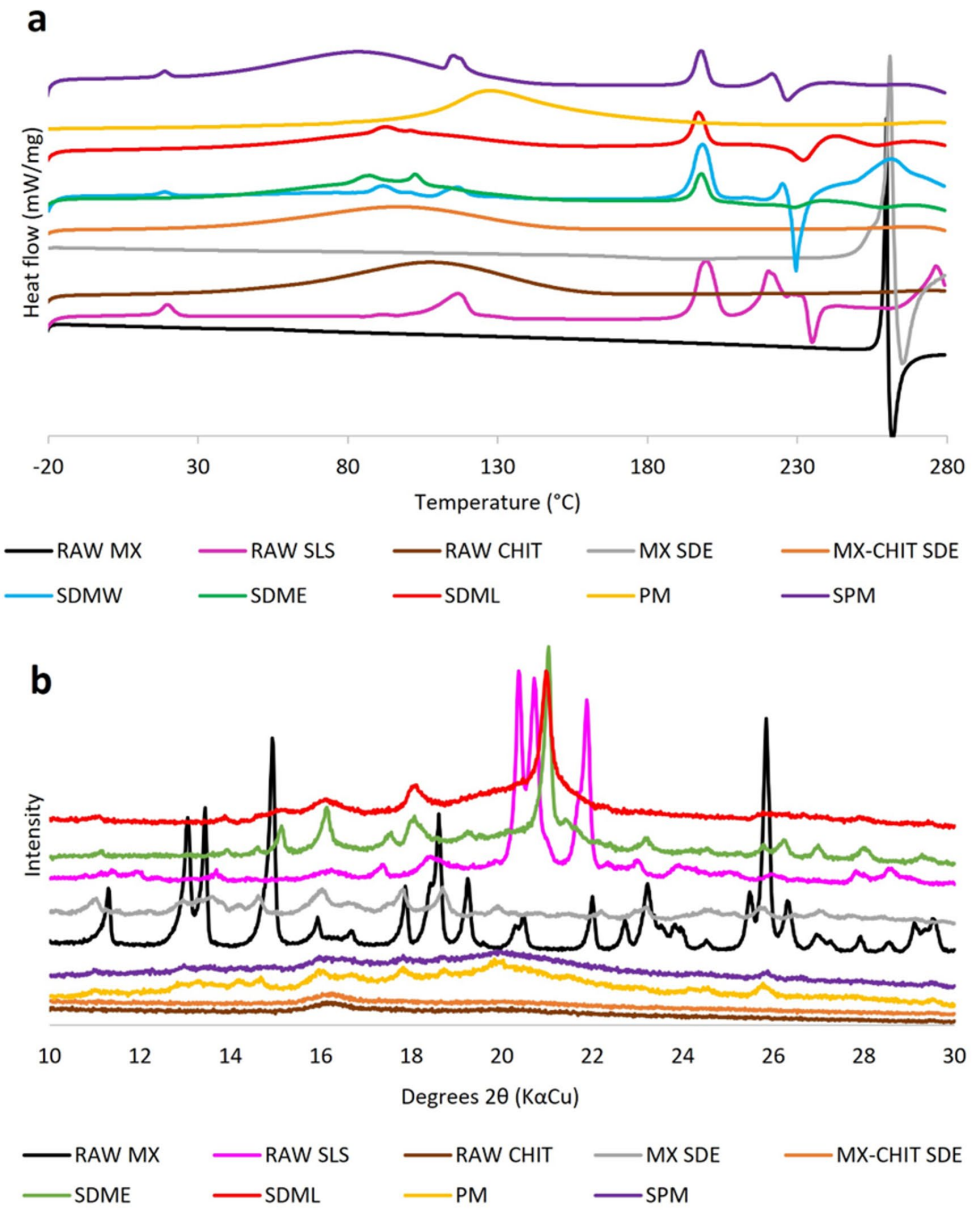
(a) DSC thermograms and (b) XRPD patterns of raw substances, spray-dried samples, and physical mixtures.

DSC thermograms (Fig. 3a) confirmed the crystalline nature of RAW MX, with a sharp melting endotherm at 259.9 °C (enthalpy ΔH 88.7 J·g^–1^), corresponding to the Form I^25,47,48^. MX SDE exhibited a similar melting point (261.8 °C) with slightly reduced enthalpy, suggesting partial amorphization. In contrast, no distinct MX melting peak was observed in the SDMs or physical mixtures, likely due to the low drug content and/or amorphization during processing.

Amorphous CHIT displayed a broad dehydration peak at ~ 99 °C, while SLS showed characteristic dehydration and melting transitions. These features were retained in the SDMs, indicating the presence of intact excipients. The absence of MX melting in PM and SPM, where no thermal stress was applied, supports the hypothesis that low drug concentration limits detection sensitivity rather than indicating structural transformation. Moreover, CHIT and SLS were well detectable in SDMs. As described by Silveira et al.^50^, small shifting in characteristics might result from the interaction of substances during the spray drying; here, the CHIT dehydration could be facilitated by the lowering of solid-liquid interfacial tension by SLS^51^.

XRPD patterns (Fig. 3b) confirmed the crystalline Form I of RAW MX, with sharp Bragg peaks at characteristic 2θ values (e.g., 11.3°, 13.0°, 17.9°, 25.8°)^25,47,52^. MX SDE showed reduced peak intensity and slight shifts (e.g., 19.9°, 27.0°), suggesting partial conversion to a more soluble amorphous form and polymorph, possibly Form III. In MX-CHIT SDE, only the amorphous halo of CHIT was visible, indicating complete amorphization of MX. As mentioned above, this sample reached even 100% *m*_rel_ within 80 s (Fig. 2b) with the highest detected *r*_*rel*_.

SDME and SDML exhibited low-intensity peaks at ~ 21.0° 2θ, attributed to SLS, which crystallizes in a monoclinic lattice^53^. These peaks are consistent with hexagonally packed polymethylene chains^54,55^, suggesting a structural rearrangement of SLS during spray drying^46^. No distinct MX peaks were detected, supporting its amorphous or molecularly dispersed state.

In PM and SPM, weak MX Form I peaks were present, confirming the crystalline nature of MX in these mixtures. However, their low intensity again reflects the limited drug content.

**Fig. 4:**
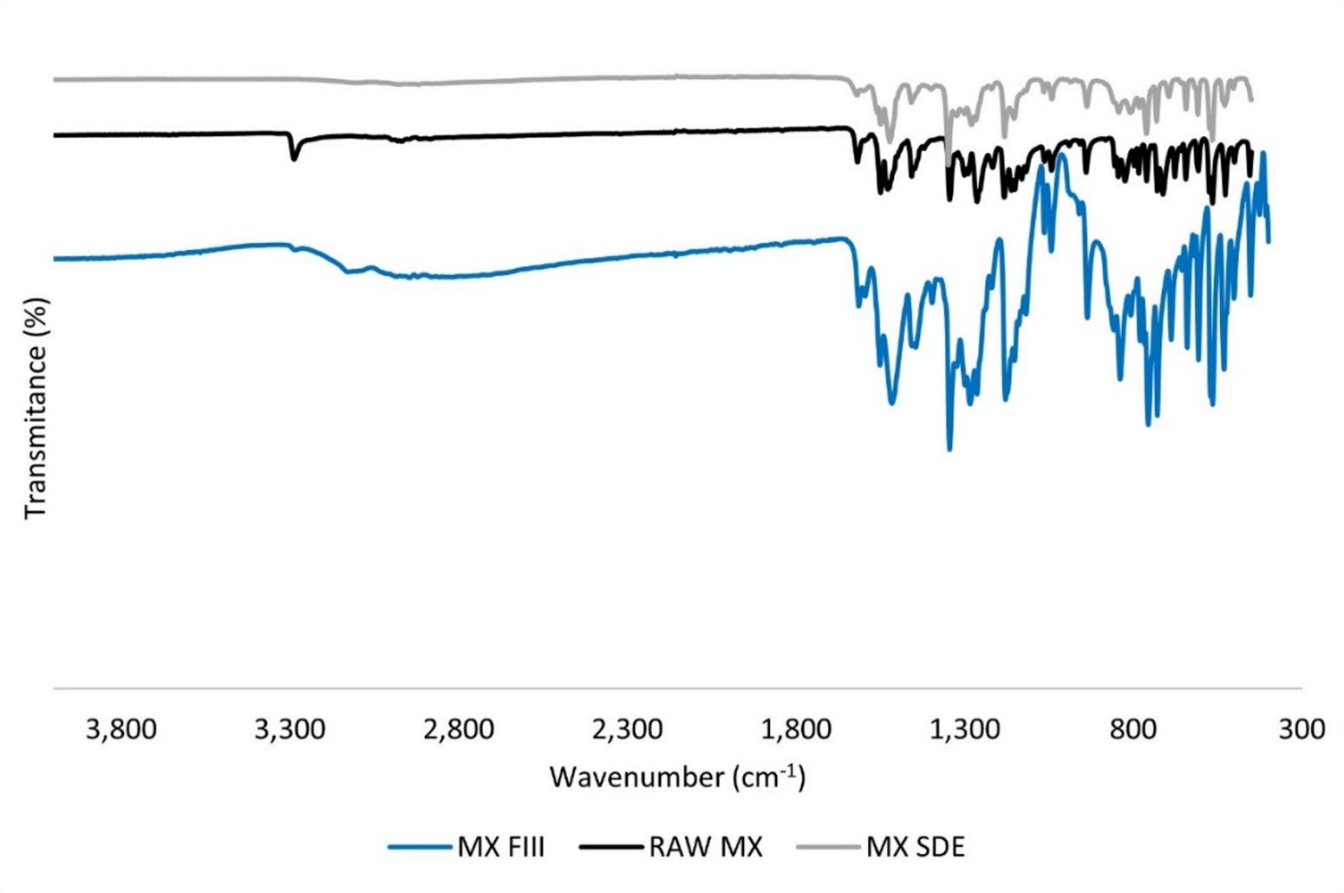
FTIR spectra of meloxicam Form I (RAW MX), meloxicam Form III (MX FIII), and meloxicam spray dried from ethanol (MX SDE).

FTIR analysis was employed to further investigate the solid-state form of MX in the MX SDE sample (Fig. 4). The spectrum of RAW MX (Form I) displayed characteristic N–H stretching vibrations at 3,288 cm^–1^ and 825 cm^–1^, consistent with literature data for the stable crystalline form. In contrast, these bands were absent in the MX SDE spectrum, which instead exhibited new bands at 3,100 cm^–1^ and 1,400 cm^–1^, features associated with the metastable Form III^47,52^.

The shift of the N–H stretching band to lower wavenumbers suggests stronger intermolecular hydrogen bonding, indicative of structural rearrangement. These findings support the partial transformation of MX to Form III and/or an amorphous state in the MX SDE sample, in agreement with XRPD results. The improved dissolution of MX SDE (Fig. 2b) can thus be attributed to both the presence of a more soluble polymorph and the reduced particle size (Table 2), which enhances surface area and dissolution kinetics.

#### Molecular imaging and spatial distribution

**Fig. 5:**
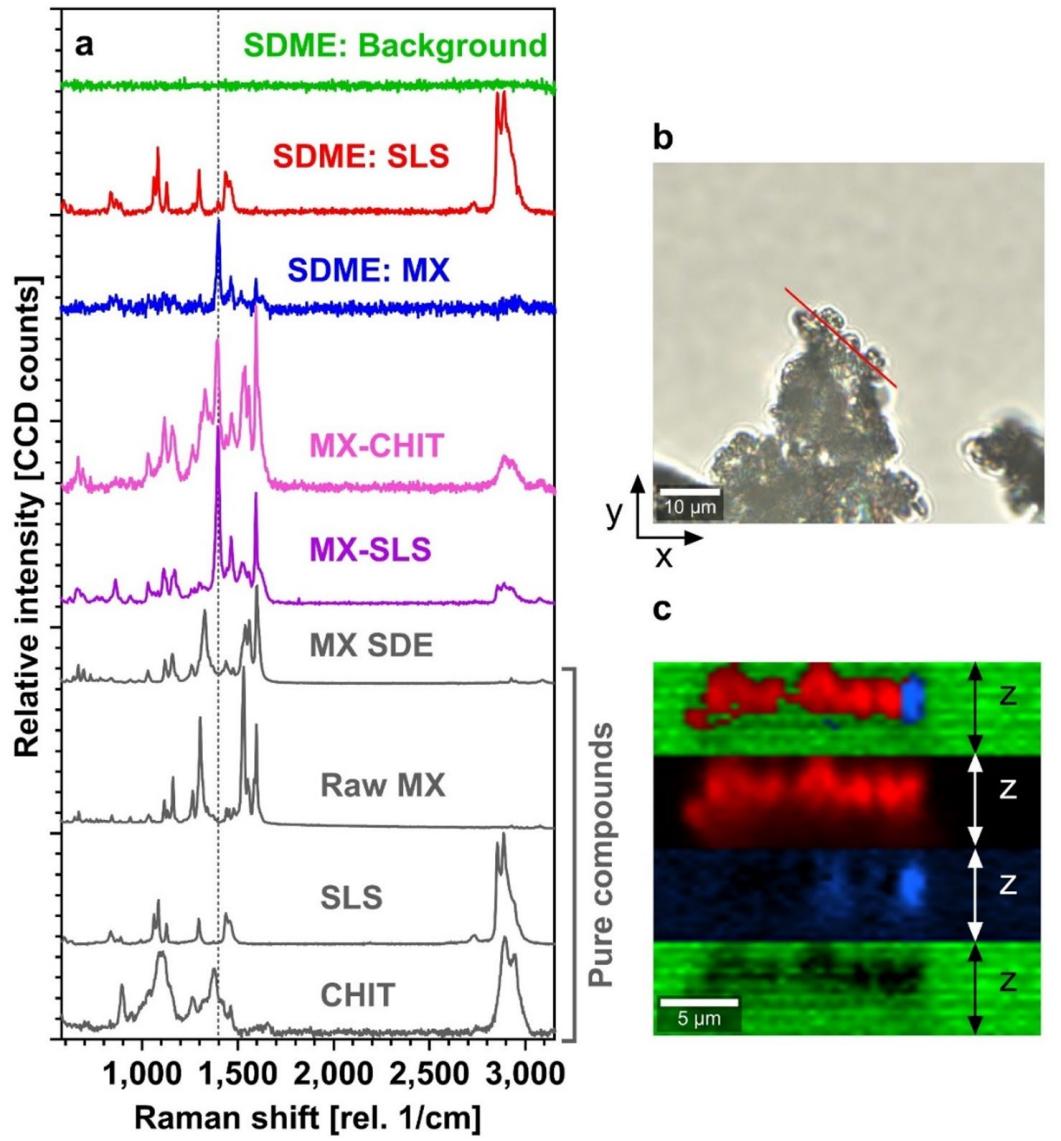
(a) Raman spectra of pure components and MX SDE (grey), binary mixtures: MX-CHIT and MX-SLS, and components identified in the SDME depth profile: MX SDME-component (blue line), SLS SDME-component (red line) and background (green line). Dashed line at 1,398 cm^− 1^ is discussed in the text. Spectra are scaled and shifted along y axis for clarity. (b) A microscopic picture of the SDME particle with the indicated position of the depth profile measurement. (c) The SDME Raman depth profile with the colour scale corresponding to the Raman spectra in panel a).

To further elucidate the spatial organization and molecular interactions within the spray-dried systems, confocal Raman microscopy was employed. Raman spectra of RAW MX (Form I) exhibited characteristic bands at 1,162 cm^–1^ and 1,596 cm^–1^ (Fig. 5a), consistent with its crystalline structure^12,48,52,56^. In MX SDE, these bands were present but attenuated, and a new band at ~ 1,300 cm^–1^ suggested partial conversion to Form III.

In the SDME sample, a microscopic image of the particle (Fig. 5b) indicates the position of the depth profile measurement. In the sample, Raman mapping revealed distinct domains of MX and SLS, indicating partial phase separation (Fig. 5c). The CHIT signal was not detected, likely due to its low Raman scattering efficiency. Notably, a prominent band at 1,398 cm^–1^ was observed, absent in RAW MX but present in both MX-SLS and MX-CHIT binary systems spray-dried from ethanol (Fig. 5a). This band is attributed to interactions involving the enolic –OH group of MX, potentially through hydrogen bonding or partial deprotonation. The interaction with CHIT is supported by its basic microenvironment (pH ~ 8.2–8.4), which may transiently promote deprotonation of MX. Similar spectral shifts have been reported for MX in micellar systems and polymer matrices^12,51,57^. Thus, the 1,398 cm^–1^ band likely reflects combined effects of hydrogen bonding with CHIT and SLS, rather than salt formation, which was not observed in 1:1 SLS–CHIT mixtures^51^.

**Fig. 6:**
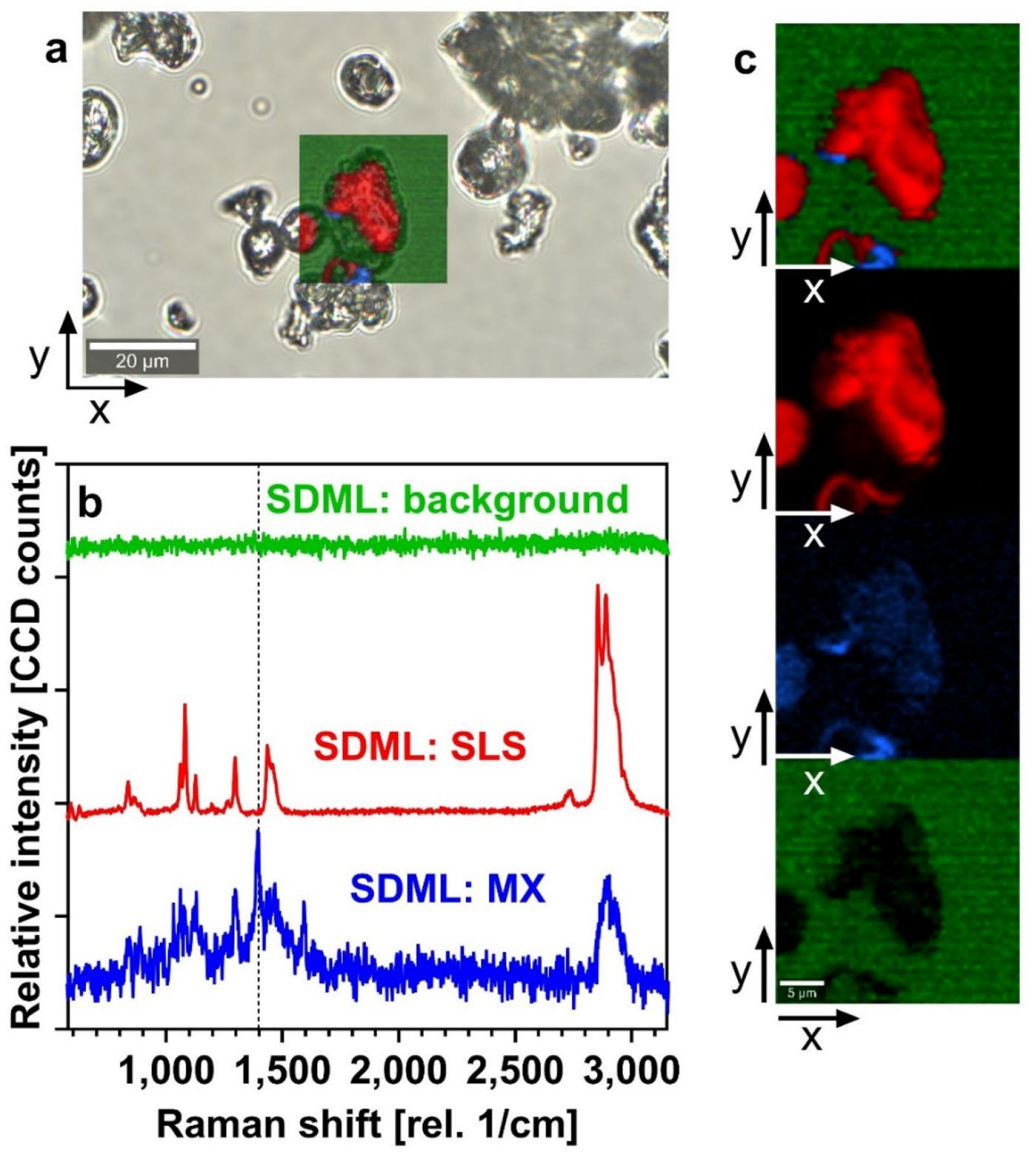
(a) A microscopic picture of the SDML particle with the indicated position of the area scan. (b) Raman spectra of components identified in the SDML area scan: MX SDML-component (blue line), SLS SDML-component (red line) and background (green line). Dashed line at 1,394 cm^− 1^ is discussed in the text. Spectra are scaled and shifted along y axis for clarity. (c) The SDML Raman maps with the colour scale corresponding to the Raman spectra in panel b).

In SDML, Raman area scans confirmed the presence of MX on the surface of CHIT particles (Fig. 6a and c), supporting the hypothesis of deagglomeration and improved dispersion. The band at 1,394 cm^–1^ (Fig. 6b), analogous to that in SDME, further indicated MX–excipient interactions. Depth profiling confirmed the close contact of MX and the solubilizing SLS resulting in the higher wettability and better enhanced availability for the dissolution media and rapid dissolution observed in vitro (Fig. 2a, b).

#### Pharmaceutical applicability

**Fig. 7:**
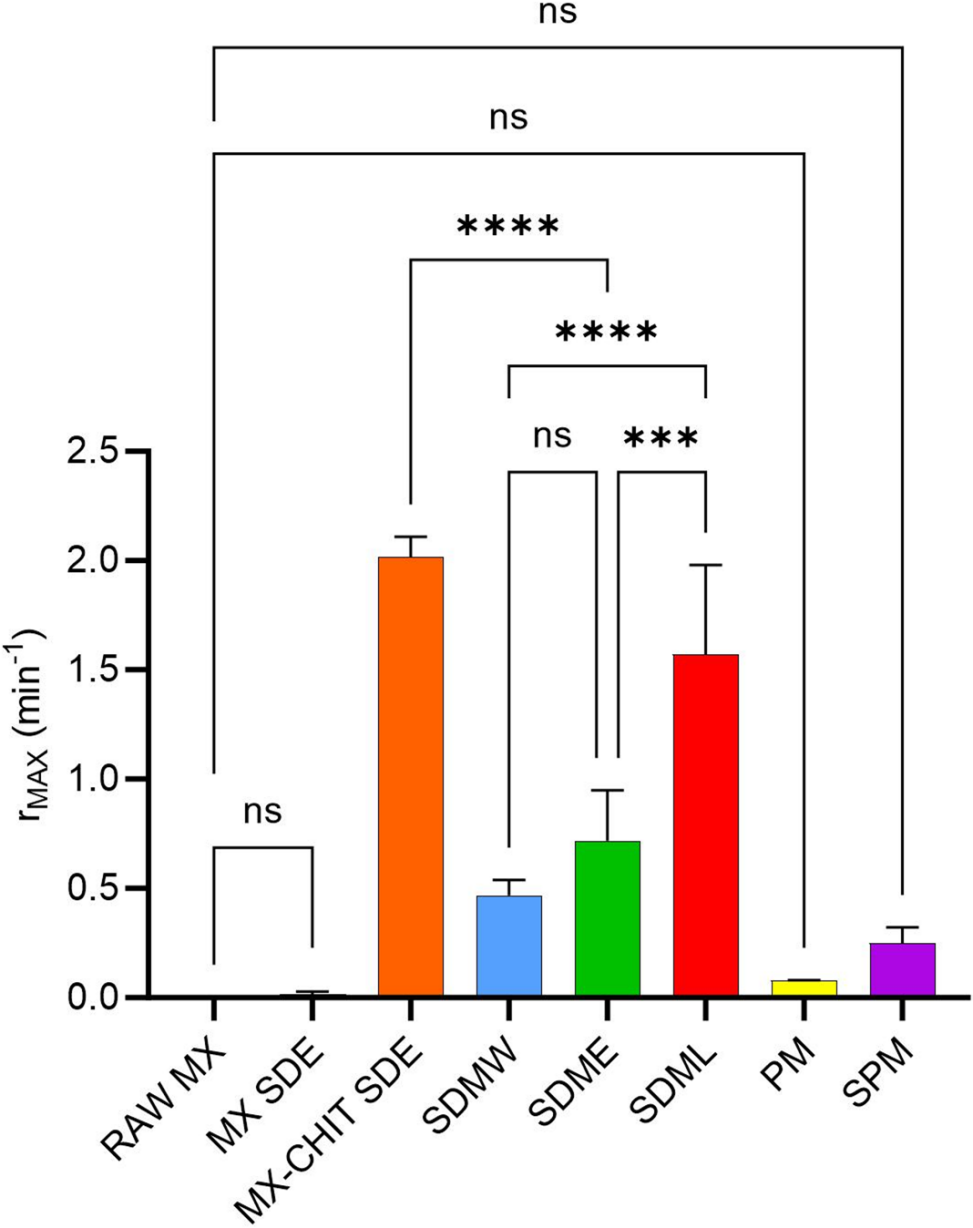
Comparison of significancy between the maximum relative dissolution rate *r*_MAX_ (min^− 1^) of meloxicam in the samples (RAW meloxicam, spray dried samples and physical mixtures). Asterisks indicate a comparison significancy evaluated by ANOVA (α = 0.05). Stars mark significant difference (*** = *p* = 0.0001; **** = *p* < 0.0001) while *ns* stands non-significant difference (*p* > 0.9999).

The co-spray drying of MX with CHIT and SLS demonstrated a clear synergistic effect, significantly enhancing the dissolution performance of this poorly soluble BCS Class II drug. This formulation strategy combines the solubilizing capacity of SLS with the hydrophilic and carrier properties of CHIT, offering a promising platform for improving oral bioavailability. The efficiency of this approach is best illustrated by the comparison of maximum relative dissolution rates (*r*_*MAX*_) across all samples (Fig. 7). All spray-dried mixtures (SDMs) exhibited markedly higher *r*_*MAX*_ values compared to RAW MX, confirming the superior dissolution kinetics achieved through co-processing.

Although physical mixtures (PM and SPM) showed modest increases in the total amount of dissolved MX (*m*_*rel*_), their *r*_*MAX*_ values did not differ significantly from RAW MX. This highlights the importance of the co-processing technique itself, rather than the mere presence of excipients. The *r*_*MAX*_ parameter, being independent of the dissolution time point, provides a robust metric for evaluating the peak dissolution performance of each formulation.

Importantly, the successful use of a medium-scale spray dryer (SDML) with a significantly higher production yield (62%) compared to mini-scale systems demonstrates the scalability of this approach. This represents a promising step toward industrial application, supporting the feasibility of translating the formulation strategy into pharmaceutical manufacturing.

## Conclusions

This study demonstrated that co-spray drying of meloxicam (MX) with chitosan carrier (CHIT) and sodium lauryl sulphate solubilizer (SLS) significantly enhances the dissolution rate of this poorly soluble model drug. The ternary systems produced by both mini- and medium-scale spray dryers outperformed RAW MX and physical mixtures in terms of dissolution efficiency, reaching up to 80% of the relative amount released within 5 min. This was approximately three times higher compared to the physical mixtures containing the same components.

Mechanistic analysis revealed that the improvement in dissolution is driven by multiple factors summarized as: particle size reduction, micellar solubilization by SLS, homogeneous distribution of MX on the CHIT surface, and partial amorphization/conversion to Form III of MX depending on the solvent used. Combination of spectroscopic methods (DSC, XRPD, FTIR), together with Raman mapping confirmed partial structural changes in MX, including indications of transformation into more soluble forms and molecular-level interactions with both CHIT and SLS within the internal structure of SDM samples.

Three key mechanisms were identified: (1) adsorption of MX onto the surface of larger hydrophilic CHIT particles, reducing agglomeration of micronized drug; (2) surface hydrophilization by SLS, improving wettability; and (3) increased overall matrix hydrophilicity, facilitating contact between MX and the dissolution medium and leading to faster and more extensive drug release.

These findings confirm the synergistic effect of combining a hydrophilic carrier and a surfactant via co-spray drying and highlight the potential of this formulation strategy for improving the performance of BCS class II drugs. The use of a simple method of co-spraying the excipient (CHIT) dispersion with the drug solution, without the need to influence the pH to achieve chitosan dissolution, is an additional benefit of this preformulation work. However, it should be noted that MX detection was challenging due to its low concentration in the samples, and further optimization of component ratios is warranted in future studies.

## Supplementary Information

Below is the link to the electronic supplementary material.

Supplementary Material 1

## Data Availability

The datasets generated and/or analysed during the current study are available from the corresponding author on reasonable request.
