# Supplementary material for: Mechanistic insights into dissolution enhancement of co-spray dried meloxicam with chitosan and solubilization agent
Source: Sci Rep. 2025 Dec 14;16:1690. doi: 10.1038/s41598-025-31245-1 (PMC12800161; doi:10.1038/s41598-025-31245-1)
Supplement: Supplementary file 1 — Supplementary Material 1 [file 41598_2025_31245_MOESM1_ESM.docx]

**Supplementary**

Supplementary Table S1. Operating parameters of spray drying process

| **Process parameters** | **Experimental conditions** |
| --- | --- |
| **Mini Büchi spray dryer** | |
| Nozzle diameter | 1.4 mm, two-fluid nozzle |
| Air flow rate | 200 – 800 L/h |
| Aspirator | 100 % |
| Inlet temperature | 170 °C |
| Outlet temperature | 130 – 150 °C |
| Pump | 7 % |
| **Mini Büchi spray dryer with inert loop and dehumidifier** | |
| Nozzle diameter | 0.7 mm, two-fluid nozzle |
| Gas flow rate | 0.75 L/h |
| Aspirator | 100 % |
| Inlet temperature | 85 °C |
| Outlet temperature | 52 – 76 °C |
| Pump | 40 % |
| **Laboratory spray dryer** | |
| Nozzle speed | 40,000 rpm, rotary nozzle |
| Air flow rate | 100,000 L/h |
| Inlet temperature | 170 °C |
| Pump performance | 10 % (27 mL/min for water) |
